# Effect of elevation on composition and diversity of fungi in the rhizosphere of a population of *Deyeuxia angustifolia* on Changbai Mountain, northeastern China

**DOI:** 10.3389/fmicb.2023.1087475

**Published:** 2023-04-20

**Authors:** Xin Sui, Mengsha Li, Beat Frey, Guanhua Dai, Libin Yang, Mai-He Li

**Affiliations:** ^1^Engineering Research Center of Agricultural Microbiology Technology, Ministry of Education, Heilongjiang University, Harbin, China; ^2^Heilongjiang Provincial Key Laboratory of Ecological Restoration and Resource Utilization for Cold Region, School of Life Sciences, Heilongjiang University, Harbin, China; ^3^Swiss Federal Institute for Forest, Snow and Landscape Research WSL, Birmensdorf, Switzerland; ^4^School of Forestry, Northeast Forestry University, Harbin, China; ^5^Institute of Nature and Ecology, Heilongjiang Academy of Sciences, Harbin, China; ^6^Research Station of Changbai Mountain Forest Ecosystems, Chinese Academy of Sciences, Erdaobaihe, China; ^7^Key Laboratory of Geographical Processes and Ecological Security in Changbai Mountains, Ministry of Education, School of Geographical Sciences, Northeast Normal University, Changchun, China; ^8^School of Life Sciences, Hebei University, Baoding, China

**Keywords:** altitudinal gradients, dominant fungi, Illumina sequencing, richness of soil fungi, soil microbial community, soil physicochemical properties

## Abstract

Soil fungi are a key component of terrestrial ecosystems and play a major role in soil biogeochemical cycling. Although the diversity and composition of fungal communities are regulated by many abiotic and biotic factors, the effect of elevation on soil fungal community diversity and composition remains largely unknown. In this study, the soil fungal composition and diversity in *Deyeuxia angustifolia* populations along an elevational gradient (1,690 m to 2020 m a.s.l.) were assessed, using Illumina MiSeq sequencing, on the north-facing slope of the Changbai Mountain, northeastern China. Our results showed that soil physicochemical parameters changed significantly along with the elevational gradients. The Ascomycota and Basidiomycota were the most dominant phyla along with the gradient. Alpha diversity of soil fungi decreased significantly with elevation. Soil nitrate nitrogen (NO_3_^−^-N) was positively correlated with fungal richness and phylogenetic diversity (PD), indicating that soil nitrate nitrogen (NO_3_^−^-N) is a key soil property determining fungal community diversity. In addition to soil nitrate content, soil pH and soil moisture were the most important environmental properties determining the soil fungal diversity. Our results suggest that the elevational changes in soil physicochemical properties play a key role in shaping the community composition and diversity of soil fungi. This study will allow us to better understand the biodiversity distribution patterns of soil microorganisms in mountain ecosystems.

## 1. Introduction

Mountains have attracted increasing curiosity of ecologists because of their high sensitivity to global climate change (Frey et al., 2016). Elevational gradients in the high mountains are often characterized by dramatic changes in the abiotic and biotic factors within short geographical distances (Rime et al., 2016; Donhauser and Frey, 2018; Adamczyk et al., 2019; Zhang et al., 2021), including vegetation, soil physicochemical properties, precipitation, temperature and illumination (Shen et al., 2013; Lin et al., 2017; Zhang et al., 2022), which may lead to significant changes in soil microorganisms within short distance. For example, soil bacterial community diversity varied significantly with elevation, while soil fungal community diversity did not change across an elevational gradient of 400 m (Yu et al., 2019), but both the soil fungal and bacterial community diversities changed significantly with elevation across an elevational gradient of 1,500 m in the Tibetan Plateau (Han et al., 2022).

In recent years, many microorganisms have been discovered in a variety of cold environments (Sahay et al., 2013; Shivaji et al., 2013; Prasad et al., 2014; Rime et al., 2016; Donhauser et al., 2020; Zong and Fu, 2021; Zhang et al., 2022). On the Changbai Mountain, northeastern China, many studies have investigated the structure and function of microbial community in forest soils (Zhou, 2006; Wang et al., 2013; Li et al., 2017; Ping et al., 2017), but no studies have investigated the elevational patterns of fungi in grassy soils on the Changbai Mountain. *Deyeuxia angustifolia* is a typical grass in the mountain ecosystems that plays an important role in biogeochemistry (Sui et al., 2021; Weng et al., 2022). This species is distributed on the Chnagbai Mountain from 1,690 m to 2020 m a.s.l., which provides an ideal field platform to study the diversity of soil fungi with an elevational gradient but within the same herbaceous plant population.

Soil fungi are key components, playing an important role in biogeochemical cycling and litter decomposition of terrestrial ecosystem, and are closely related to soil properties and aboveground vegetation community characteristics (Wang et al., 2013; Ping et al., 2017; Ni et al., 2018; Hanif et al., 2019; Ren et al., 2019; Sui et al., 2021; Yang et al., 2021; Zhou et al., 2021). Soil fungal community diversity showed a significant relationship with soil pH (Fouts et al., 2012; Zhou et al., 2021), C/N ratio, soil temperature and soil organic carbon (Ping et al., 2017; Sui et al., 2021; Zhou et al., 2021; Deng et al., 2023). Different ecosystems with different characteristics determine the characteristics of soil fungal communities (Shi et al., 2014). The traditional explanation for this phenomenon is that aboveground vegetation affects soil fungal communities by altering the physicochemical properties of the soils. Soil, vegetation, and climatic factors change gradually with increasing elevation, suggesting that the fungal community may vary along with elevational gradients on mountains (Tedersoo et al., 2016).

The Changbai Mountain is an important gene pool of biodiversity in Northeast China (Xue and Tisdell, 2001; Tang et al., 2011). Its rich species diversity has made it a research hotspot. As one of the main vegetation species on Changbai Mountain, *D. angustifolia* is distributed from 1,690 to 2020 m and is indispensable for the protection of ecosystem functions. According to previous reports, *D. angustifolia* in the alpine tundra are invaded from lower elevations due to climate change (Zong et al., 2013, 2014), indicating that the soil fungal community in the alpine tundra ecosystem could also correspondingly change. Because soil fungi play an important role in litter degradation and nutrient cycling (Frey et al., 2016; Deng et al., 2023), the changes in fungal community composition and diversity caused by *D. angustifolia* invasion may directly affect the ecosystem structure and function. Therefore, understanding the changes in soil fungal community composition and diversity under *D. angustifolia* at different elevations can help us predict changes in ecosystem structure and function following *D. angustifolia* invasion. Unfortunately, there are no comprehensive studies on soil fungi in *D. angustifolia* population along elevational gradients.

To comprehensively understand these changes, we investigated the composition and diversity of soil fungal community in *D. angustifolia* population at 1,690, 1,800, 1,910, and 2,020 m above sea level (a.s.l.) along an elevational gradient, using Illumina Miseq sequencing, on the Chnagbai Mountain. We hypothesize that the soil fungal composition and diversity in *D. angustifolia* population change significantly along the elevational gradient, because the soil environmental characteristics that shape the soil fungal composition and diversity change with increasing elevation. Therefore, the objectives of this study were (1) to compare the fungal diversity and community composition in *D. angustifolia* population in response to elevation, and (2) to evaluate the relationships between soil fungal communities and soil physicochemical properties across the elevational gradient.

## 2. Materials and methods

### 2.1. Research site

This study was performed on the Changbai Mountain (126^°^55′-129^°^00′E, 41^°^23′-42^°^36’N) in northeastern China. The local climate is a typical continental temperate monsoon climate with a daily average temperature of 5.9°C during the growing season (June to September). The average annual precipitation during the growing season can reach 958 mm. The mean annual precipitation and temperature is approximately 600 mm and 4°C, respectively.

To study the elevational pattern of soil fungal composition and diversity, we selected pure *D. angustifolia* population at 1,690, 1,810, 1,910, and to 2020 m a.s.l. along an elevational gradient on the north-facing slope of the Changbai Mountain. During October 1 to 7, 2018, three independent plots (10 m × 10 m) were set up in *D. angustifolia* population at each elevation. Ten to fifteen soil samples (0 ~ 20 cm organic layer) were sampled and pooled for each plot, using a sterile soil drill (5 cm in diameter, 20 cm deep). After removing the surface litter and humus layer, approximately 1 kg soils for each plot were collected. The soil samples were sieved (2 mm mesh) to remove stones, visible roots and residues and other debris, and divided into two sub-samples: one stored at −80°C for sequencing, and the other one stored at 4°C for soil physicochemical properties.

### 2.2. Measurements of soil chemical properties

A soil-water (deionized water) (1:2.5 w/v) suspension was shaken for 30 min prior to measuring the pH with a pH meter (Thermo Scientific Orion 3-Star Benchtop, Cambridge, United Kingdom). Soil moisture content (SMC) was measured by comparing the fresh wet weight with the dry weight after drying at 120°C for 24 h. Soil organic carbon (SOC) and the total nitrogen (TN) content were measured using an elemental analyzer (Elementar, Langenselbold, Germany). Ammonium (NH_4_^+^-N) and nitrate (NO_3_^−^-N) nitrogen content were measured using a continuous flow analysis system (SKALAR SAN++, Breda, the Netherlands). The total phosphorus (TP) content was measured using a spectrophotometer, and available phosphorus (AP) content was measured using the colorimetric method upon extraction with 0.5 M NaHCO_3_. The total potassium (TK) content was measured by digesting the soil with concentrated hydrofluoric acid, and available potassium (AK) content was extracted by acetic acid and ammonium leaching method. The extracted TK and AK content were determined using inductively coupled plasma atomic emission spectrometry (ICP-AES-7500, Shimadzu, Japan). Soil microbial biomass C (MBC) and biomass N (MBN) content were measured with a TOC analyzer (TOC-LCPH, Shimadzu, Japan). Soil mechanical compositions (Sand, Silt, Clay) were determined according to the method of Zhang et al. (2021). Three independent replicates per sample were performed for all the soil physicochemical properties.

### 2.3. Soil DNA extraction and ITS rRNA sequencing

Using the MOBIO Power Soil Extraction Kit (Mo Bio Laboratories, Carlsbad, CA, United States), soil total DNA was extract from 1 g of fresh soil according to the manufacturer’s instructions. The DNA was diluted in TE buffer (DNA Elution Solution-Ultra Pure Water). The DNA quantity and quality were detected using a NanoDrop ND-1000 spectrophotometer (Thermo Scientific, United States).

Fungal ITS rRNA region was amplified using primers ITS1 (5′-CTTGGTCATTTAGAGGAAGTAA-3′) and ITS2 (5′- GCTGCGTTCATCGATGC -3′) (Fouts et al., 2012). A 6-bp barcode sequence unique to each sample was added to the primers for distinguishing multiple samples. The PCR reaction was performed in triplicate in a 25 μL mixture containing 2.5 μL of TransStart Buffer, 2 μL of dNTPs, 1 μL of each primer (10 ng/μL), and 30 ng of template DNA. The PCR conditions were as follows: pre-denaturation at 94°C for 5 min, 30 cycles of dunaturation at 94°C for 30 s, 55°C for 30 s, 72°C for 45 s, and a final extension at 72°C for 10 min. The PCR products were inspected by 2% agarose electrophoresis, and were purified using the AxyPrep DNA purification kit. Three independent PCR replicates per sample and then three PCR samples were pool at equal amount and PE300 paired-end sequenced on the Illumina Miseq v3 platfrom (2 × 300 bp). The raw sequences were uploaded to the Sequence Read Archive (SRA) database and accession number was SUB10527794.

### 2.4. Bioinformatics and statistical analysis

Sequences were analyzed using QIIME (version 1.8^1^) software on the Allwegene bioinformation cloud platform.^2^ The original PE reads were quality filtering following criteria: if the mean score < 20 or the length < 200 bp, and the ambiguities sequence were removed. The forward and reverse reads merged using PEAR software (version 0.9.8). The chimeras removed using Usearch (version 7.1).^3^Operational taxonomic units (OTU) were generated at a similarity level of 97% using the UPARSE algorithm (Edgar, 2013). Taxonomic analysis was performed on the representative sequences of OTU, with a confidence threshold of 0.7, and the UNITE 8.2 fungi database was used for comparison.^4^

Before further analysis, the sequences were normalized according to the lowest number of sequences for a single sample. The sequences occurring <1% were classified into “others.” Alpha diversity indexes (Richness, Chao1, Shannon index, PD=Phylogenetic diversity) were calculated in QIIME1 based on OTU table. Principal coordinate analysis (PCoA) was finished using R software (v.3.2.5, R Development Core Team, 2016) “Vegan” package based on Bray–Curtis dissimilarity at OTU level. Rarefaction curve was finished using R software (v.3.2.5, R Development Core Team, 2016) “microeco” package. Redundancy analysis (RDA) was performed using R software (v.3.2.5, R Development Core Team, 2016) “microeco” package based on OTU table and soil physicochemical parameters. Fungal functional guilds were assigned by using FUNGuild v1.0 and the differences of guilds among elevations were performed by one-way analysis of variance (ANOVA), Duncan test (*p* < 0.05). One-way analysis of variance (ANOVA) was used to detect the difference of soil physicochemical parameters among elevations using SPSS software (version 22.0). Pearson correlation analysis among fungal community composition (both phyla and genera) and soil physicochemical properties was also performed using SPSS software (version 22.0). Permutational multivariate analysis of variance (PERMANOVA) was used to test the differences in soil fungal composition among the four elevations, using Bray-curtis distance matrices (i.e., the adonis2 function of the vegan package) (Zhong and Fu, 2022).

## 3. Results

### 3.1. Soil physicochemical properties

All soil physicochemical parameters, except SOC and sand content, were significantly different (*p* < 0.05) among the four elevations (Table 1). Soil pH, and the content of SMC, nitrate nitrogen and available potassium declined with increasing elevation, while other soil physicochemical properties did not show a clear tendency (Table 1). Silt ranged from 0.5% (1,910 m a.s.l.) to 1.6% (2,020 m a.s.l.), and the clay ranged from 4.3% (1,690 m a.s.l.) to 5.1% (1,910 m a.s.l.).

**Table 1:** Soil physicochemical characteristics along an elevational gradient on the Changbai Mountains, northeastern China.

Properties^1^	1,690 m a.s.l.	1,800 m a.s.l.	1,910 m a.s.l.	2,020 m a.s.l.
SMC	44.0 ± 1.17^a^	37.6 ± 0.89^b^	33.4 ± 1.06^b^	36.0 ± 5.29^b^
pH	5.5 ± 0.15^a^	4.6 ± 0.15^b^	4.6 ± 0.09^b^	4.7 ± 0.08^b^
NH_4_^+^-N (mg/kg)	0.7 ± 0.13^c^	2.0 ± 0.06^a^	0.8 ± 0.03^c^	1.4 ± 0.12^b^
NO_3_^−^-N (mg/kg)	1.9 ± 0.07^a^	0.4 ± 0.02^b^	0.2 ± 0.01^c^	0.2 ± 0.00^c^
SOC (g/kg)	10.3 ± 0.63^a^	10.1 ± 0.36^a^	9.4 ± 0.60^a^	9.5 ± 0.67^a^
TN (g/kg)	9.9 ± 0.08^c^	19.3 ± 0.86^a^	11.0 ± 0.51^c^	14.6 ± 0.83^b^
TK (g/kg)	3.1 ± 0.28^b^	4.7 ± 0.19^a^	4.6 ± 0.40^a^	3.3 ± 0.13^b^
AK (mg/kg)	17.7 ± 0.63^b^	24.0 ± 0.60^a^	13.9 ± 0.60^d^	15.1 ± 0.33^c^
AP (mg/kg)	11.7 ± 0.31^c^	17.0 ± 0.22^a^	15.1 ± 0.22^b^	10.3 ± 0.53^d^
MBC (mg/kg)	516.5 ± 4.90^b^	569.4 ± 5.73^b^	675.5 ± 3.68^a^	527.1 ± 4.11^c^
MBN (mg/kg)	69.0 ± 2.05^d^	89.3 ± 2.94^a^	84.7 ± 2.05^ab^	76.8 ± 2.45^c^
Sand (%)	94.7 ± 4.50^a^	96.7 ± 6.24^a^	95.2 ± 4.92^a^	95.2 ± 4.92^a^
Silt (%)	1.1 ± 0.08^c^	0.6 ± 0.05^d^	0.5 ± 0.03^d^	1.6 ± 0.04^a^
Clay (%)	4.3 ± 0.22^c^	4.8 ± 0.25^ab^	5.1 ± 0.08^a^	4.5 ± 0.34^bc^

1 Values represent means ± standard deviations (*n* = 3). Different letters indicate significant (*P* < 0.05) differences between individual means assessed by one-way ANOVA followed by Tukey’s HSD *post-hoc* testing. SMC, soil moisture content; NH_4_^+^-N, Ammonium nitrogen; NO_3_^−^-N, Nitrate nitrogen; SOC, soil organic cabon; TN, total nitrogen; TK, Total potassium; AK, Effective potassium; AP, Effective phosphorus; MBC, Microbial biomass carbon; MBN, Microbial biomass nitrogen.

### 3.2. Rarefaction curve and fungal diversity

**Figure 1:**
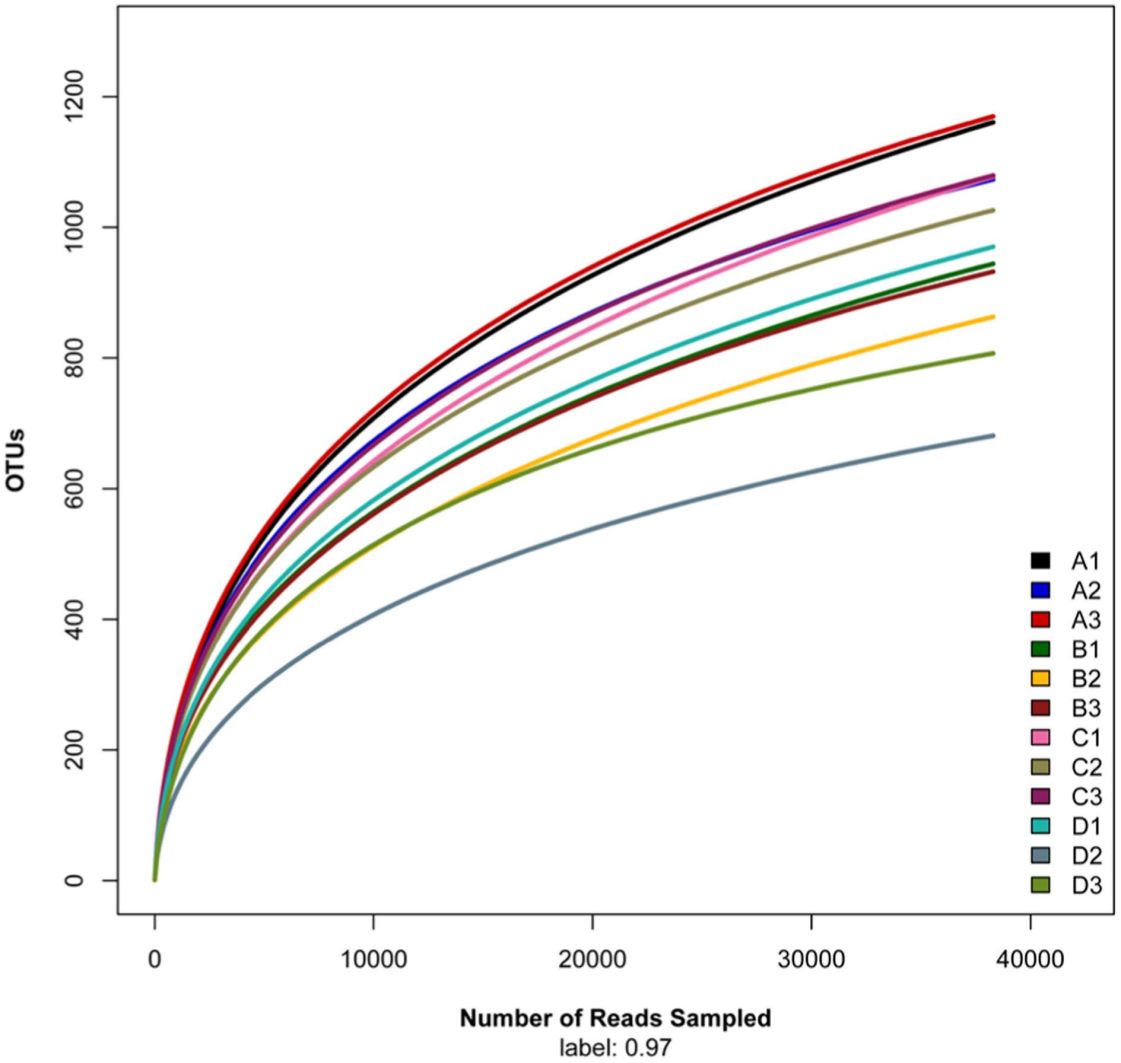
Rarefaction curve of fungal sequences in soils along an elevational gradient on the Changbai Mountain, northeastern China. Note A (1–3) is 1,690 m a.s.l.; B (1–3) is 1,800 m a.s.l.; C (1–3) is 1910 m a.s.l.; D (1–3) is 2020 m a.s.l.

The rarefaction curve (Figure 1) tended to flatten, indicating that the sequencing number was sufficient and reasonable to cover the fungal communities. The Chao1, richness, phylogenetic diversity (PD), and Shannon index of the soil fungi were significant different (one way ANOVA, *p* < 0.01) among elevations along the elevational gradient (Table 2). The alpha diversity indices including Chao1, richness, PD_whole_tree, and Shannon-Wiener decreased from 1,690 m to 1800 m a.s.l., then increased to 1910 m a.s.l., followed by decreasing tendency up to 2,020 m a.s.l.

**Table 2:** Fungal alpha diversity along an elevational gradient on the Changbai Mountain, northeastern China^1^.

Elevation (m a.s.l.)	Chao1 index	Richness index	Phylogenetic diversity index	Shannon index
1,690	1464.09 ± 105.47a	1097.83 ± 51.39a	200.07 ± 10.77a	6.64 ± 0.14a
1,800	1268.34 ± 32.80abc	877.53 ± 41.70abc	164.99 ± 4.86bc	6.15 ± 0.27ab
1,910	1425.33 ± 73.89ab	1023.50 ± 29.97ab	187.86 ± 5.24ab	6.74 ± 0.05a
2,020	1087.65 ± 223.13c	792.10 ± 139.36c	151.65 ± 19.67c	5.72 ± 0.56b

^1^Values represent means ± standard deviations (*n* = 3). Different letters indicate significant (*P* < 0.05) differences between individual means assessed by one-way ANOVA followed by Tukey’s HSD *post-hoc* testing.

Pearson correlation analysis showed that the soil fungal alpha diversity (richness and PD index) were significantly positively correlated with the content of soil NO_3_^−^-N, but significantly negatively correlated with the content of soil NH_4_^+^-N and TN (Table 3). PD index of soil fungi was significantly positively correlated with soil pH, while the Shannon-Wiener index of soil fungi was significantly negatively correlated with the content of soil TN (Table 3).

**Table 3:** Pearson’s rank correlation coefficients between fungal alpha-diversity and soil physicochemical characteristics.

	pH	NH_4_^+^-N	NO_3_^−^-N	SMC	TN	TK	AK	AP
Chao1	0.31	−0.40	0.45	−0.24	−0.47	0.15	−0.01	−0.16
Richness	0.44	−0.53^*^	0.57^*^	−0.20	−0.59^**^	0.00	−0.06	−0.23
PD	0.47^*^	−0.57^*^	0.60^**^	−0.28	−0.62^**^	0.02	−0.09	−0.28
Shannon	0.23	−0.42	0.35	−0.15	−0.49^*^	0.17	−0.06	−0.08

* Correlation is significant at the 0.05 level (one-tailed).

** Correlation is significant at the 0.01 level (two-tailed).

SMC, soil moisture content; NH_4_^+^-N, Ammonium nitrogen; NO_3_^−^-N, Nitrate nitrogen; SOC, soil organic cabon; TN, total nitrogen; TK, Total potassium; AK, Effective potassium; AP, Effective phosphorus; MBC, Microbial biomass carbon; MBN, Microbial biomass nitrogen.

### 3.3. Composition of the soil fungal community

**Figure 2:**
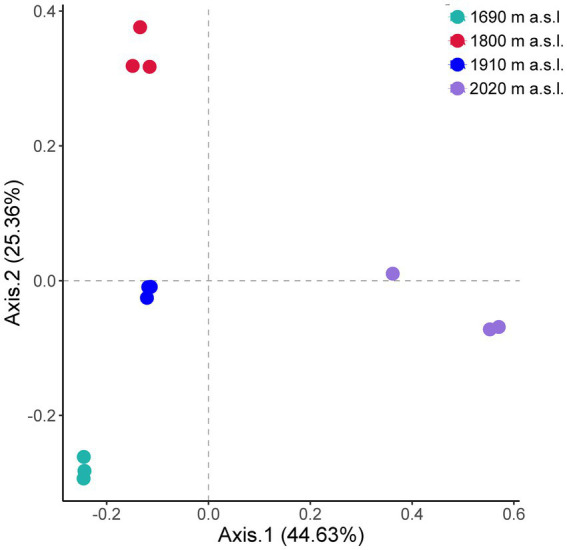
Principal coordinate analysis (PCoA) of fungal communities along an elevational gradient on Changbai Mountain, northeastern China. The dominant plant species at the sites were *D. angustifolia.* Beta-Diversity index was calculated at the OTU level (97%).

The ordination showed that the fungal communities were clearly separated by elevation (PCo1, 44.63% and PCo2, 25.36%) (Figure 2). The soil fungal beta diversity differed significantly among elevations (Figure 2, Anosim *R* = 0.97, *p* < 0.01). Moreover, there were significant differences in species composition between any two out of the four elevations (Supplementary Table S2, *p* < 0.05).

**Figure 3:**
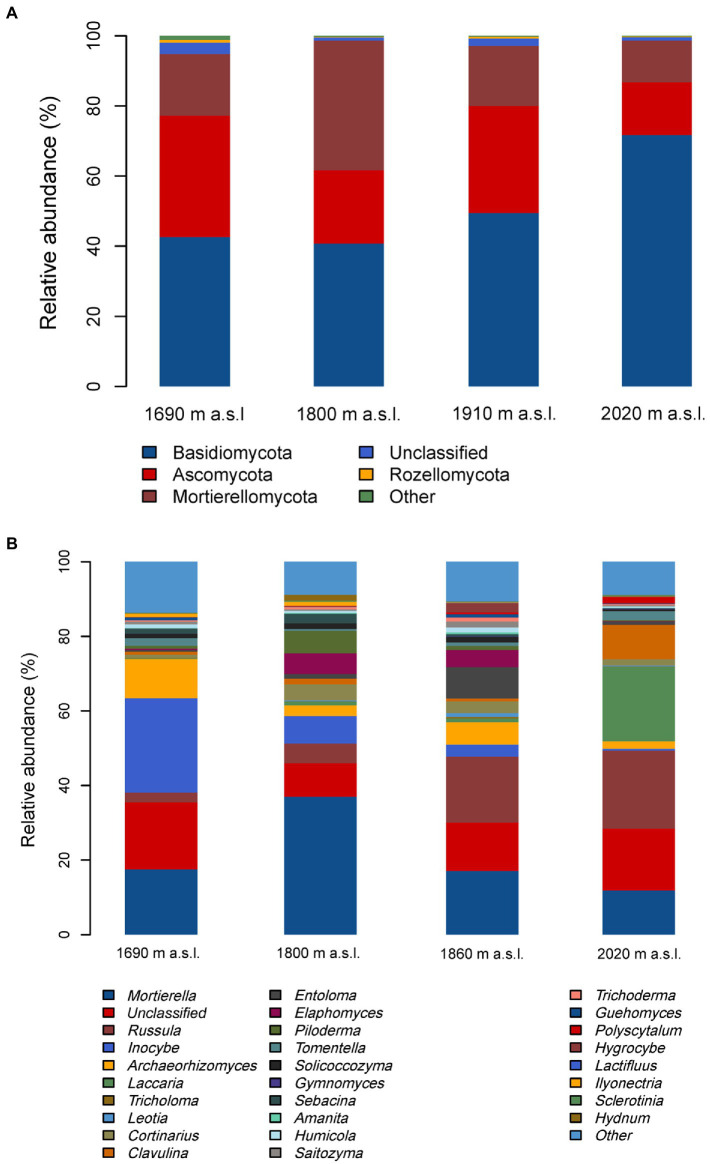
Relative abundance of the dominant fungal phyla **(A)** and genera **(B)** in soils along an elevational gradient on the Changbai Mountain, northeastern China.

The soil fungal community composition (both at the phyla and genera level) was significantly different among the four elevations (Figure 3 and Supplementary Tables S1, S3). All the obtained sequences belonged to 8 phyla. The prevailing phyla was Basidiomycota (53% relative abundance), Ascomycota (26%), and Mortierellomycota (19%) across all the soil samples (Figure 3A). The relative abundance of these dominant fungal phyla changed remarkably with elevation (Supplementary Table S1), showing that the relative abundance of Basidiomycota had an increased tendency but Ascomycota showed a decreased tendency with increasing elevation (Figure 3A and Supplementary Table S1).

The dominant genera with a relative abundance of >1% were *Mortierella* (18.7% relative abundance), *Russula* (13.3%), *Inocybe* (7.7%), *Archaeorhizomyces* (4.7%), *Laccaria* (4.2%), *Tricholoma* (3.2%), *Leotia* (2.5%), *Cortinarius* (2.5%), *Clavulina* (2.4%), *Entoloma* (2.1%), *Elaphomyces* (2.0%), *Piloderma* (1.6%), *Tomentella* (1.3%), *Solicoccozyma* (1.1%), *Gymnomyces* (1.1%), *Sebacina* (1.0%) (Figure 3B). *Russula* was most abundant at the highest site (2,020 m a.s.l.), while *Inocybe* was most abundant at the lowest elevational site (Figure 3B). Similar to the phyla, the relative abundance of these most dominant fungal genera also changed remarkably with increasing elevation (Supplementary Table S3).

### 3.4. Relationships between fungal community and soil properties

**Figure 4:**
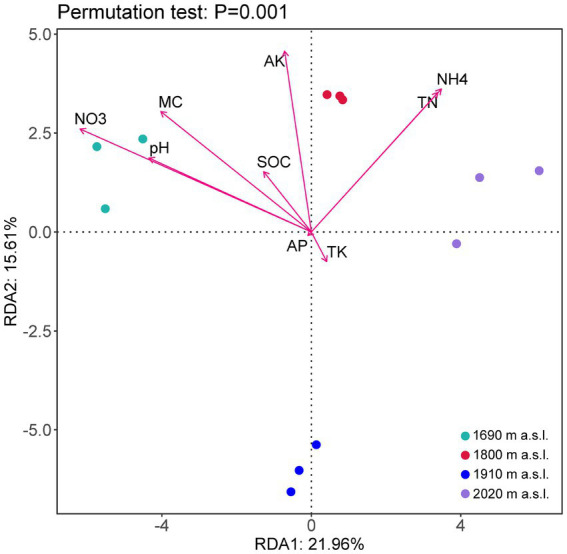
Redundancy analysis (RDA) of soil fungal community structures and environmental characteristics (arrows) at different elevations.

RDA revealed that soil properties (i.e., NH_4_^+^-N, NO_3_^−^-N, TN, SOC, MC, pH, AK, TK, and TP) were the key environmental factors that shaped the soil fungal community (Figure 4). The first two axes of the RDA accounted for 37.57% of the total variance. As shown in Figure 4, MC (*p* < 0.05), pH (*p* < 0.05), NH_4_^+^-N (*p* < 0.05), NO_3_^−^-N (*p* < 0.05), TN (*p* < 0.05) significantly influenced the fungal community. The soil fungal community structure at 2,020 m a.s.l. was significantly positively correlated with NH_4_-N and TN, while that at 1,690 m a.s.l. and 1,800 m a.s.l. was significantly positively correlated with soil moisture content, pH, AK and NO_3_^—^N (Figure 4).

At the phyla level, the abundance of phyla was closely correlated with some certain, phyla-specific soil physicochemical factors (Table 4). For the two dominant phyla, for example, the abundance of Ascomycota was significantly negatively correlated with the content of soil TN, NH_4_^+^-N, and AP (Table 4), while the abundance of Basidiomycota was significantly negatively correlated with soil TK (Table 4).

**Table 4:** Pearson’s rank correlations between the relative abundances of dominant fungal taxa and soil physicochemical variables.

	SMC	pH	NH_4_^+^-N	NO_3_^−^-N	SOC	TN	TK	AK	AP
Phylum	–	–	–	–	–	–	–	–	–
Basidiomycota	−0.29	−0.26	0.04	−0.45	0.34	0.06	−0.49^*^	−0.37	−0.02
Ascomycota	0.25	0.47	−0.64^**^	0.49^*^	−0.20	−0.61^**^	0.19	−0.25	−0.05
Chytridiomycota	0.30	0.48^*^	−0.32	0.59^*^	−0.28	−0.40	−0.28	−0.04	−0.34
Glomeromycota	0.67^**^	0.84^**^	−0.53^*^	0.96^**^	−0.05	−0.55^*^	−0.50^*^	0.13	−0.40
Mortierellomycota	0.11	−0.14	0.58^*^	0.07	−0.27	0.53^*^	0.55^*^	0.74^**^	0.12
Mucoromycota	0.79^**^	0.88^**^	−0.35	0.95^**^	−0.08	−0.44	0.36	0.32	−0.35
Rozellomycota	−0.06	0.21	−0.47	0.19	−0.37	−0.37	0.08	−0.49^*^	−0.13
Genus	–	–	–	–	–	–	–	–	–
*Inocybe*	0.77^**^	0.83^**^	−0.38	0.92^**^	0.19	−0.42	−0.49^*^	0.32	−0.13
*Mortierella*	0.11	−0.14	0.5708^*^	0.07	−0.27	0.53^*^	0.55^*^	0.74^**^	0.12
*Archaeorhizomyces*	0.59^*^	0.78^**^	−0.67^**^	0.82^**^	−0.31	−0.72^**^	−0.17	−0.09	−0.42
*Russula*	−0.59^**^	−0.55^*^	−0.05	−0.76^**^	0.03	−0.01	0.06	−0.70^**^	0.02
*Tomentella*	0.33	0.56^*^	−0.40	0.44	−0.41	−0.48^*^	−0.53^*^	−0.36	−0.92^**^
*Solicoccozyma*	0.12	−0.05	−0.01	0.15	−0.30	−0.09	0.53^*^	0.20	0.14
*Guehomyces*	−0.11	−0.08	−0.36	−0.03	0.21	−0.33	−0.15	−0.29	0.28
*Sebacina*	0.41	0.08	0.52^*^	0.26	0.19	0.46	0.13	0.93^**^	0.36
*Myrothecium*	0.41	0.40	−0.26	0.56^*^	−0.11	−0.34	−0.39	0.05	−0.24
*Cortinarius*	−0.30	−0.64^**^	0.64^**^	−0.52^*^	0.02	0.63^**^	0.64^**^	0.53^*^	0.52^*^
*Ilyonectria*	0.58^*^	0.38	0.32	0.60^**^	−0.13	0.22	0.05	0.85^**^	−0.04
*Humicola*	0.07	0.09	−0.49^*^	0.13	0.03	−0.46	0.17	−0.26	0.10
*Saitozyma*	−0.25	−0.08	−0.58^*^	−0.08	0.01	−0.57^*^	0.29	−0.51^*^	0.23
*Clavulina*	−0.23	−0.12	0.22	−0.22	−0.20	0.17	−0.35	−0.16	−0.57^*^

* Correlation is significant at the 0.05 level (one-tailed).

** Correlation is significant at the 0.01 level (two-tailed).

SMC, soil moisture content; NH_4_^+^-N, Ammonium nitrogen; NO_3_^−^-N, Nitrate nitrogen; SOC, soil organic cabon; TN, total nitrogen; TK, Total potassium; AK, Effective potassium; AP, Effective phosphorus; MBC, Microbial biomass carbon; MBN, Microbial biomass nitrogen.

At the genus level, the abundance of genus was also closely correlated with some certain, genus-specific soil physicochemical factors (Table 4). For instance, the abundance of *Ilyonectria*, *Inocybe* and *Archaeorhizomyces* was significantly positively correlated with SMC, while the abundance of *Russula* was significantly negatively correlated with the content of SMC (Table 4).

### 3.5. Fungal functional guilds

The dominated functional groups of fungi were ectomycorrhizal, Endophyte, Undefined Saprotroph, Plant Pathogen, Animal Pathogen, Wood Saprotrophc, Lichenized, Ericoid Mycorrhizal and Arbuscular Mycorrhizal (Supplementary Table S4). Except Ericoid Mycorrhizal, Lichenized and Plant Pathogen, other soil fungal functional groups were significantly different among elevations along the elevational gradient (Supplementary Table S4). The absolute abundance of Arbuscular Mycorrhizal and Lichenized was highest at 1,690 m a.s.l., and the absolute abundance of Animal Pathogen, Ericoid Mycorrhizal and Wood Saprotroph was highest at 1,810 m a.s.l. while the absolute abundance of Ectomycorrhizal was highest at 2020 m a.s.l. (Supplementary Table S4).

## 4. Discussion

### 4.1. Changes in soil physicochemical properties with elevation

In this study, we found significant differences in soil physicochemical properties among elevations along the elevational gradient from 1,690 m to 2020 m a.s.l. on the Changbai Mountain (Table 1), this is consistent with the results of Zong et al. (2014) who investigated the soil physicochemical properties in *D. angustifolia* population along elevational gradients on the same mountain. The climate changes dramatically with increasing elevation on mountains, which causes markedly changes in soil environment such as biogeochemical cycling and soil nutrients (Zong et al., 2014). In our study, for example, the soil water content and pH value were significantly higher at 1,690 m than at 2,020 m a.s.l., which indicates that soil moisture holding capacity decreases with the invasion of *D. angustifolia.* We found a significant lower value of soil organic carbon at higher elevation (2,020 m) and at lower elevation (1,800 m), which is consistent with results of previous studies (Meng et al., 2018; Luo et al., 2020).

### 4.2. Changes in soil fungal alpha diversity with elevation

Soil fungi play an important role in biogeochemical cycling and ecological process (Buee et al., 2009; Russo et al., 2012), but previous studies have focused on bacterial diversity and composition, with only a few focusing on fungi in mountain ecosystems. Therefore, we have only limited information about changes in fungi with elevation (Djukic et al., 2010; Adamczyk et al., 2019; Zhou et al., 2021). Our results showed that the alpha diversity (Shannon index, Chao1 index, Richness and PD index) of soil fungi changed significantly with elevation, showing that the alpha diversity decreased with increasing elevation (Table 3). However, this pattern does not seem to be widespread. The distribution pattern of soil fungal diversity in the literature showed declining, humped, U-shaped, or no change with increasing elevation. Yang et al. (2017) reported that soil fungal diversity decreased monotonically from 700 m to 2,600 m a.s.l. across various ecosystems on the Changbai Mountain. Ping et al. (2017) reported that soil fungal diversity in *Pinus koraiensis* forest showed a hollow curve’s pattern along an elevational gradient from 699 m to 1,044 m a.s.l. on the Changbai Mountain.

Our study indicated that the alpha diversity of soil fungi showed a decreasing trend with increasing elevation. Shen et al. (2014) showed that the Chao1 index of soil fungi on the Changbai Mountain was not correlated with elevation, but it had a strong correlation with soil pH. Ni et al. (2018) found that soil fungal Chao1 index increased with increasing elevation, and soil C/N was the most important environmental factor determining the soil fungal Chao1 index along an elevational gradient from 2,000 m to 2,500 m a.s.l. on the Changbai Mountain. However, Zhang et al. (2022) found that both the Chao1 and Shannon index of soil fungi decreased significantly from 2,785 m a.s.l. to 4,578 m, and the climate and soil properties had opposite effects on them in Tibetan Plateau.

In our present study, soil TN, NH_4_^+^-N, and NO_3_^−^-N were significantly correlated with fungal richness (*r* = −0.53, *p* < 0.05; *r* = 0.57, *p* < 0.05; *r* = −0.59, *p* < 0.01) along the elevational gradient. As the soil N content decreased with increasing elevation (Zong et al., 2014), resulting thus in a reduction in soil fungal diversity found in the present study. Similar studies have also demonstrated that soil nutrients are an important environmental factor affecting the distribution pattern of soil fungal diversity (Newsham et al., 2016; Yang et al., 2017).

### 4.3. Fungal compositions

In our present study, the dominant phyla are Basidiomycota and Ascomycota, which is consistent with findings of previous studies on fungal composition along elevational gradients on Changbai Mountain. However, the elevational patterns of Basidiomycota and Ascomycota differed among studies. Our study found that Basidiomycota showed an increasing trend, while Ascomycota showed a decreasing trend with elevation from 1,690 m to 2,020 m a.s.l. However, Shen et al. (2014) and Ni et al. (2018) found that Basidiomycota showed an overall increasing trend, while Ascomycota’s did not change or tended to decrease with elevation on Changbai Montain. Ping et al. (2017) found that the abundance of Basidiomycota first decreased from 699 m to 937 m a.s.l., then increased from 937 m to 1,177 m a.s.l., while the abundance of Ascomycota increased from 699 m to 937 m a.s.l., and then decreased from 937 m to 1,177 m a.s.l. Yu et al. (2019) and Zhang et al. (2021) found that Ascomycota and Mortierellomycota are the predominant fungi in Tibetan grassland communities. This difference in dominant phyla may be closely related to the fact that the vegetation (or plant ecosystem) and soil physicochemical properties differ significantly between the two regions (Jin et al., 2018; Zong and Fu, 2021).

Soil fungal composition is affected by multiple factors such as vegetation composition, soil physicochemical properties, and microclimate along elevational gradients (Ni et al., 2018). A previous study carried ou on the Changbai Mountain found that the aboveground vegetation composition was closely related to the fungal composition, and dominant plant species significantly affected the fungal composition (Ni et al., 2018). In our present study, the dominant Basidiomycota and Ascomycota were mainly affected by soil physicochemical properties because the vegetation population (i.e., *D. angustifolia* population) did not change along with the elevational gradient. The fungal Basidiomycota and Ascomycota are involved in the soil organic metabolism (Luo et al., 2021) and thus their abundances were significantly determined by soil organic matter content as a result of decomposition of plant residues (Li et al., 2019).

Our results also showed that the functional guilds of soil fungi at high elevation were mainly ectomycorrhizal fungi and plant pathogens. Ni et al. (2018) also reported that the dominant functional fungi were ectomycorrhizal fungi in the alpine tundra on the Changbai Mountain. Similarly, Timling et al. (2014) reported that the dominant functional guilds in the arctic were the ectomycorrhizal fungi. However, in this study, the alpine tundra population of *D. angustifolia* is an invasive plant, and the effect of the original tundra species on the soil has not completely replaced by the invasive plant, so that the soil fungal functions were still ectomycorrhizal fungi and plant pathogens.

## 5. Conclusion

The present study revealed that a small elevational difference on mountains may lead to marked difference in soil physicochemical properties, fungal diversity, and community composition. Our results showed that fungal alpha diversity was higher at lower elevations, which may be a result of higher soil nutrient levels at lower elevations. The phyla of Ascomycota and Basidiomycota, as well as the genera of *Mortierella* and *Russula* dominated the soil fungal communities across the entire elevational gradient. Generally, Basidiomycota increased but Ascomycota decreased with increasing elevation. The changes in soil pH and nutrients were the most important soil environment factors leading to changes in soil fungal beta diversity. Our results highlight the different patterns of fungal communities across elevational gradients, and further elucidate the variation in fungal community composition and ecological functions in temperate mountain ecosystems.

## Data availability statement

The data presented in the study are deposited in the NCBI Sequence Read Archive repository, accession number SUB10527794.

## Author contributions

XS and ML designed and performed the experiment and prepared this manuscript. BF and M-HL revised this manuscript and language editing. GD and LY helped to do the experiment and finish the bioinformatic analysis. All coauthors contributed to manuscript editing, read, and agreed to the published version of the manuscript.

## Funding

This work was funded by the Natural Sciences Foundation of Heilongjiang Province (LH2020C088), Heilongjiang Province Postdoctoral Research Start-up Fund Project (LBH-Q21167), Outstanding Youth Foundation of Heilongjiang University (JCL202006), the China Scholarship Council Visiting Scholar Program (201908230401), and the Basic Scientific Research of Provincial Higher Education Institutions in Heilongjiang Province of 2022. Heilongjiang Provincial Ecological Environmental Protection Research Project (HST2022ST008) and the central government guides local science and technology development special projects (ZY20B15).

## Conflict of interest

The authors declare that the research was conducted in the absence of any commercial or financial relationships that could be construed as a potential conflict of interest.

## Publisher’s note

All claims expressed in this article are solely those of the authors and do not necessarily represent those of their affiliated organizations, or those of the publisher, the editors and the reviewers. Any product that may be evaluated in this article, or claim that may be made by its manufacturer, is not guaranteed or endorsed by the publisher.

## Footnotes

^1^ http://qiime.org

^2^ http://218.2.224.234

^3^ http://drive5.com/aparse

^4^ https://unite.ut.ee/
