# Supplementary material for: Effect of elevation on composition and diversity of fungi in the rhizosphere of a population of Deyeuxia angustifolia on Changbai Mountain, northeastern China
Source: Front Microbiol. 2023 Apr 20;14:1087475. doi: 10.3389/fmicb.2023.1087475 (PMC10231489; doi:10.3389/fmicb.2023.1087475)
Supplement: Supplementary file 1 [file Table_1.doc]

Supplementary Material

# Supplementary Figures and Tables

**Table S1**. Relative abundance of the dominant fungal phyla in *D. angustifolia* forest soils along an altitudinal gradient in the Changbai Mountains, northeastern China.

| Elevation (m) | Basidiomycota (%) | Ascomycota (%) | Mortierellomycota (%) |
| --- | --- | --- | --- |
| 1690 | 43.7±0.03b | 34.3±0.02a | 18.7±0.03b |
| 1810 | 41.7±0.04b | 21.7±0.02bc | 37.3±0.06a |
| 1910 | 49.3±0.01b | 30.3±0.03ab | 17.3±0.02b |
| 2020 | 72.0±0.08a | 15.0±0.02c | 12.3±0.06b |

^1^Values represent means ± standard deviations (n=3). Different letters indicate significant (*P*<0.05) differences between individual means assessed by one-way ANOVA followed by Tukey’s HSD post-hoc testing.

Table S1 The permutational multivariate analysis of variance (i.e. the ‘adonis2’ of vegan-package) of soil fungal community composition between any two latitudes in Changbai mountain.

| Latitudes | | distance | Community composition | | |
| --- | --- | --- | --- | --- | --- |
|  |  |  | *F* | *R*^2^ | *p* |
| 1690 | 1800 | Bray-Curtis | 27.37 | 0.87 | 0.1 |
| 1690 | 1910 | Bray-Curtis | 28.85 | 0.88 | 0.1 |
| 1690 | 2020 | Bray-Curtis | 25.78 | 0.86 | 0.1 |
| 1800 | 1910 | Bray-Curtis | 34.15 | 0.90 | 0.1 |
| 1800 | 2020 | Bray-Curtis | 24.83 | 0.86 | 0.1 |
| 1910 | 2020 | Bray-Curtis | 25.84 | 0.87 | 0.1 |

**Table S3**. Relative abundance of the most abundant fungal genera in *D. angustifolia* forest soils along an altitudinal gradient in the Changbai Mountains, northeastern China.

| Genus (%) | 1690 (m) | 1810 (m) | 1910 (m) | 2020 (m) |
| --- | --- | --- | --- | --- |
| *Mortierella* | 17.6±3.2bc | 37.0±5.6a | 17.1±2.1bc | 11.9±5.8bc |
| *Russula* | 2.6±0.3c | 5.3±0.8c | 17.7±0.4b | 21.0±2.6a |
| *Inocybe* | 25.3±2.6a | 7.4±0.8b | 3.2±0.4c | 0.5±0.4c |
| *Archaeorhizomyces* | 10.6%±1.8a | 2.9±0.3cd | 6.0±0.6b | 2.0±1.2d |
| *Laccaria* | 0.1±0.0b | 1.0±0.2b | 1.0±0.2b | 19.9±6.9a |
| *Tricholoma* | 0.0±0.0b | 0.0±0.0b | 0.4±0.0b | 0.2±0.1b |
| *Leotia* | 0.0±0.0c | 0.3±0.2bc | 1.0±0.1b | 0.2±0.3c |
| *Cortinarius* | 0.9±0.2d | 4.3±0.8a | 3.1±0.7b | 1.8±0.3cd |
| *Clavulina* | 0.9±0.1b | 1.5±0.2b | 0.8±0.2b | 9.2±3.5a |
| *Entoloma* | 0.4±0.1c | 1.3±0.3b | 8.4±0.5a | 0.8±0.4bc |
| *Elaphomyces* | 0.3±0.0bc | 5.5±1.1a | 4.6±0.6a | 0.1±0.2c |
| *Piloderma* | 0.8±0.0bc | 6.1±0.6a | 1.0±0.3b | 0.3±0.3c |
| *Tomentella* | 2.1±0.3a | 0.5±0.0c | 1.1±0.1b | 2.4±0.2a |
| *Solicoccozyma* | 1.2±0.3ab | 1.5±0.3a | 1.5±0.2ab | 0.5±0.6c |
| *Gymnomyces* | 0.0±0.0b | 0.0±0.0b | 0.2±0.0b | 0.1±0.1b |
| *Sebacina* | 1.4±0.3b | 2.6±0.3a | 0.6±0.2c | 0.2±0.2c |

**Table S4**. Total numbers of sequences for the fungal functional guilds in *Deyeuxia angustifolia* forest soils along an altitudinal gradient in the Changbai Mountains, northeastern China.

| FUNGuild | 1690 m | 1810 m | 1910 m | 2020 m |
| --- | --- | --- | --- | --- |
| Animal Pathogen | 161.7±25.4b | **262.3±35.2a** | 82.7±11.67cd | 93.7±28.6c |
| Arbuscular Mycorrhizal | **223.0±63.9**a | 32.7±5.9b | 29.67±5.1b | 15.67±17.9b |
| Ectomycorrhizal | 13362.7±1132.6bc | 13744±884.0bc | 13530.3±722.8bc | **22273.0±3184.2a** |
| Ericoid Mycorrhizal | *34.3**±2.5*a | **127.4±78.3**a | 60.7±14.6a | 54.0±17.7a |
| Lichenized | **242.3±286.5**a | *20.7±0.6*a | 57.3±44.1a | 32.0±5.3a |
| Plant Pathogen | 432.0±98.8a | *224.0±169.5*a | 546.67±236.25a | 836.0±677.4a |
| Undefined Saprotroph | 3085.0±1120.9b | 1795.3±402.6bc | 2556.67±316.84bc | *1347.0±134.4*c |
| Wood Saprotroph | 76.7±54.1b | **345.7±236.6**a | 53.3±44.1b | *20.3±17.9*b |

Statistical significance (One-way ANOVA, *P*<0.05) is indicated by different superscript letters in the same row. In each column, the largest value is shown in bold and the smallest value is shown in italics.
